# Hashimoto's Disease and Thyroid Cancer in Children: Are They Associated?

**DOI:** 10.3389/fendo.2018.00565

**Published:** 2018-10-09

**Authors:** Laura Penta, Marta Cofini, Lucia Lanciotti, Alberto Leonardi, Nicola Principi, Susanna Esposito

**Affiliations:** ^1^Pediatric Clinic, Department of Surgical and Biomedical Sciences, Università Degli Studi di Perugia, Perugia, Italy; ^2^Università degli Studi di Milano, Milan, Italy

**Keywords:** anti-thyroid antibodies, Hashinoto's disease, thyroid cancer, thyroid nodules, TSH

## Abstract

Hashimoto's thyroiditis (HT) is the most common cause of thyroid disease in children and adolescents. Along with significant modifications of thyroid function, HT in pediatric age can be accompanied by relevant thyroid structural alterations. Over time, benign thyroid nodules, carcinoma and, rarely, primary non-Hodgkin lymphoma can develop. However, the relationships between HT and neoplasms are poorly defined. The main aim of this paper is to discuss what is presently known regarding the coexistence of HT and thyroid tumors. Moreover, we attempt to define the pathogenesis of cancer development in children with HT. Literature analysis showed that despite its rarity and relatively promising prognosis, thyroid cancer is associated with HT. Although not all reasons for the coexistence of these diseases are clearly defined, children with HT should be considered at higher risk for thyroid cancer development. Strict correlations between high levels of serum TSH and anti-thyroid antibodies with cancer must be remembered. The same is true for the presence of nodules, especially if multiple nodules are present and ultrasonography and thyroid fine needle aspiration cytology should be promptly used in uncertain cases.

## Introduction

Hashimoto's thyroiditis (HT), also termed chronic lymphocytic thyroiditis, is the most common cause of thyroid disease in children and adolescents. It is an autoimmune disease with an estimated prevalence in pediatrics of 1–2%, with variations according to genetic susceptibility, age and gender, ethnicity, iodine status, the presence of other autoimmune diseases or genetic syndromes and the criteria used for diagnosis (1). HT is more common in children aged 6 to 16 years, in females, in Caucasians, and in countries with iodine deficiency. Moreover, it is more frequently diagnosed in children who suffer from type 1 diabetes, coeliac disease, Addison's disease, autoimmune hypoparathyroidism, Down syndrome, Noonan syndrome, and Turner syndrome, as well as when antibody assays and thyroid fine needle aspiration cytology (TFNAC) are available (1).

At the time of diagnosis, most children with HT show few to no symptoms. A small goiter or the presence of mild clinical symptoms of hypothyroidism are observed in ~70% of the causes of hospital referrals (2). Other reasons include findings upon work-up for an unrelated problem or for one of the diseases mentioned above that pose the child at higher risk of developing HT. Thyroid function, as evidenced by blood thyroid hormone levels, is normal in up to 80% of cases (2). Only a minority has low hormone concentrations, suggesting overt hypothyroidism. In rare cases, hyperthyroidism can be demonstrated. Long-term outcomes of HT can significantly vary and are not predictable in single cases. However, both children who are initially euthyroid and those with subclinical hypothyroidism can develop overt hypothyroidism within a few years from diagnosis. Although this is more common in subclinically hypothyroid patients (3–6), conversion to Graves' disease cannot be excluded (7). The presence of goiter and elevated serum concentrations of anti-thyroglobulin antibody (TG-Ab) and anti-thyroid peroxidase antibody (TPO-Ab) at diagnosis or the progressive increase in serum TSH levels suggests an increased risk of hypothyroidism (5, 8). Finally, patients with hyperthyroidism generally become euthyroid and only occasionally develop hypothyroidism (4).

Along with significant modifications of thyroid function, HT in children can be accompanied by relevant thyroid structural alterations. Over time, benign thyroid nodules, carcinoma and, rarely, primary non-Hodgkin lymphoma can develop (9–11). However, the relationships between HT and neoplasms are poorly defined. In particular, it is not known whether HT is a predisposing factor to the development of thyroid neoplasms and whether other clinical manifestations or laboratory biomarkers can permit the early identification of HT children that are at higher risk of tumor development. The main aim of this paper is to discuss what is presently known regarding the coexistence of HT and thyroid tumors. Moreover, we attempt to define the pathogenesis of cancer development in children with HT.

## Epidemiology of pediatric thyroid cancer

Although the incidence rates of pediatric thyroid cancer have progressively increased over the past thirty years (9–11), this disease remains rare in children and adolescents compared to adults. Only 2% of the ~60,000 cases annually diagnosed in the USA regard subjects younger than 19 years of age (12). However, thyroid cancer plays a relevant role in pediatric oncology, as, in the USA, it is the eighth most common cancer diagnosed in patients aged 15–19 years and is the second most common cancer among adolescent girls (13, 14).

Along with differences in frequency compared to adult cases, pediatric thyroid cancer has several other differences regarding both histology and clinical characteristics. From a histological point of view, differentiated thyroid cancer comprises 90–95% of all childhood thyroid cancers, with papillary thyroid carcinoma (PTC) accounting for the majority of cases. In contrast, medullary thyroid cancer and undifferentiated and anaplastic forms that can be diagnosed in adults are exceptionally rare in children (15). Thyroid nodules are significantly less common in children (16, 17). However, pediatric thyroid nodules have a higher likelihood of malignancy compared to adults (16–20).

Several factors were found to be potential predictors of thyroid nodule malignancy. Microcalcifications, hypoechoic pattern, intranodular vascularization, lymph node alterations, and TSH concentration were identified by Mussa et al. (21). Papendieck et al. (22) added multinodular goiter, solid nodules, irregular margins and TSH values >2.5 mIU/L.

Finally, in contrast to adults, children with cancer at presentation have more extensive disease, with positive cervical lymph nodes and evidence of local or distant metastasis and have a higher risk of recurrence. In contrast, pediatric PTC has an excellent long-term prognosis, with 30-year survival rates of 90–99% (20, 23–26). All these findings, that are summarized in Table 1, have raised the supposition that pediatric thyroid cancer might be distinct from that of adults (27).

**Table 1:** Main characteristics of pediatric thyroid cancer.

**Characteristic**	**Comparison with adults**
Incidence rate	Significantly lower
Histology	Mainly represented by papillary thyroid cancer
	Lower frequency of thyroid nodules that have a higher likelihood of malignancy
Clinical findings	More extensive disease with positive cervical lymph nodes and evidence of local or distant metastasis
Outcome	Higher risk of recurrence
	Excellent long-term survival rate

## Coexistence of thyroid cancer and hashimoto's thyroiditis (HT) in children

Most of the studies regarding the potential association between HT and thyroid cancer development have been carried out in adults. With few exceptions (28), most have clearly demonstrated that the coexistence of HT and thyroid tumors, mainly PTC, is common and that the risk of development of thyroid cancer in patients with HT is significantly higher than that in patients without HT. Moreover, HT seemed to have a certain protective effect on the short- and long-term prognosis of cancer. In a meta-analysis of 38 articles published before September 2011, including 10,648 PTC cases (29), the frequency of HT in PTC cases was ~23%, ranging from 5 to 85%. Different diagnostic criteria for HT, various surgical procedures, and heterogeneity of enrolled patient characteristics may explain the differences. HT was more frequently observed in PTC than in benign thyroid diseases and other carcinomas (odds ratio [OR] = 2.8 and 2.4, respectively; *P* < 0.001). The association was more common in females (OR = 2.7; *P* < 0.001) and was found to have more favorable clinical and histological characteristics than PTC without HT (29). In patients with HT-associated PTC, carcinoma had no extrathyroidal extension (OR = 1.3; *P* = 0.002), no lymph node metastasis (OR = 1.3; *P* = 0.041), and a long recurrence-free survival (hazard ratio [HR] = 0.6; *P* = 0.001). Similar results were reported in a more recent meta-analysis (30). In this case, 27 studies published until December 2015, enrolling 76,281 patients and including 12,476 cases of thyroid cancer, were analyzed. The mean rate of PTC among patients with HT ranged from 1.1 to 40.1%, with variations strictly related to the methods used to diagnose thyroid cancer, being higher when diagnosis is performed with more effective radiological and laboratory methods (30). The overall pooled OR of the PTC risk for HT (HT vs. non-HT) was 2.12 (95% confidence interval [CI] = 1.78–2.52).

In children, the few available studies had even more severe limitations than those enrolling adults. They were retrospective, frequently included small numbers of children and used different criteria for the diagnosis of both HT and thyroid cancer. This precludes pooling and comparison. However, for children and adolescents, the association between HT and thyroid cancer seems relatively common. The frequency of PTC in children and adolescents with HT was found variable from 0.67 to ~3% (31–33). In patients with PTC, the prevalence of coexisting HT varied from 6.3% to more than 40% of the cases. In a recent study, in which modern histological and laboratory methods, including thyroid fine needle aspiration cytology (TFNAC), were used to diagnose HT and thyroid cancer, HT was detected in 28.7% of the 108 thyroid cancer cases (34).

However, the impact of pediatric HT on the short- and long-term prognosis of cancer was not clearly defined. In the study by Iliadou et al. (34), HT was associated with a more severe clinical manifestation of the neoplasm. Histological examination revealed that infiltration of the thyroid parenchyma, revealing invasive characteristics of the cancer, was more frequent in children with HT (74.2 vs. 48.1%; *P* = 0.024), but the final prognosis was not influenced by HT. The clinical condition of patients with or without HT was strikingly similar after 5 or 10 years of follow-up. In contrast, in the study by Ren et al. (35), in which a frequent association between HT and cancer was shown (HT in 44.2% of differentiated thyroid cancer and 41.3% of thyroid nodules), no significant differences were observed in the clinical characteristics of the thyroid tumor among children with or without HT. No differences in tumor multifocality (*P* = 0.7), tumor size (*P* = 0.09), extrathyroidal infiltration (*P* = 0.6), or metastasis (*P* = 0.34) were shown (35). However, as evidenced by Iliadou et al. (34), no effect of HT on short-term disease-free survival was shown.

## Supposed reasons for the coexistence of thyroid cancer and hashimoto's thyroiditis

Several hypotheses have been proposed to explain the potential relationship between HT and thyroid cancer (Table 2). One of them regards the role of TSH. It has been shown that the risk of thyroid malignancy is strictly related to the serum level of TSH, even with serum TSH levels within the normal range (36). In a recent prospective study, it was found that the risk of malignancy was ~3-fold higher in patients with TSH levels ≥2.26 μIU/mL than in patients with lower TSH levels (*P* = 0.001) (37). Thyroid autoimmunity that leads to higher serum TSH concentrations may partially explain the association between HT and PTC. However, it remains unclear whether TSH simply promotes the growth of a pre-existing cancer or truly causes cancer development.

**Table 2:** Hypotheses proposed to explain the potential relationship between Hashimoto's thyroiditis (HT) and thyroid cancer.

**Mechanism**	**Action**
High serum TSH level	Growth of a pre-existing cancer or cancer development induced by TSH
Chronic inflammation due to autoimmunity	Proliferation, reduction of apoptosis and angiogenesis sustained by cytokines, chemokines, and growth factors
	Facilitation of carcinogenesis programmes by extracellular matrix-modifying enzymes
Gene expression	Proinflammatory proteins produced by gene rearrangements and point mutations in proto-oncogenes increase proliferation and invasiveness of tumor cells, stimulation of angiogenesis, and reduction of anti-tumoural immune responses

*TSH, thyroid-stimulating hormone*.

A second hypothesis considers the role of chronic inflammation due to autoimmunity. HT is associated with chronic inflammation of thyroid tissue. Inflammation can increase the risk of cancer by providing bioactive molecules from cells infiltrating the tumor microenvironment (38). Cytokines, chemokines, and growth factors favor sustained proliferation, a significant reduction of apoptosis, and angiogenesis. Moreover, the production of extracellular matrix-modifying enzymes, such as metalloproteinases, is induced. This production promotes epithelial-mesenchymal transition and facilitates other carcinogenesis programmes, such as genome instability, immune evasion, and modifications of energy metabolism (39, 40). On the other hand, a complete concordance of all the markers of thyroid autoimmunity with thyroid cancer development has been reported. Boi et al. (39) carried out a retrospective analysis on 2,053 patients with single/prevalent thyroid nodules submitted to TFNAC and found that a higher prevalence of suspicious/malignant or indeterminate cytological findings was detected in patients with positive TG-Ab and thyroid microsomal antibody (TM-Ab) than in those with benign cytology. Increased independent OR for malignancy was conferred by any anti-thyroid antibody (OR 2.21; 95% CI = 1.49–3.29, *P* < 0.0001), TPO-Ab (OR 2.15; CI = 1.42–3.25, *P* < 0.0001) and TG-Ab (OR 1.67; CI = 1.05–2.67, *P* < 0.05).

A third hypothesis considers genetics. Molecular genetic studies have shown an association of HT with gene rearrangements and point mutations in the proto-oncogenes implicated in PTC, suggesting a potential interrelationship between the two diseases (18). Proinflammatory proteins (several cytokines and chemokines) induced by these mutated genes are relevant for the mobility, proliferation, survival, and invasiveness of tumor cells; stimulation of angiogenesis; and reduction of anti-tumoural immune responses. The best example in this regard is given by the chromosomal rearrangement involving the *RET* receptor tyrosine kinase gene. The rearrangement, named RET/PTC, fuses the 3′ terminal portion of *RET* coding for the tyrosine kinase domain with the 5′ terminal sequence of different unrelated genes, leading to constitutive activation of the *RET* tyrosine kinase. It is frequently identified in patients with PTC, although with significant differences according to several factors, including methodological and ethnic differences. However, one of the most important differences is age: RET/PTC rearrangements are much more frequent in younger patients with PTC, especially in children. These rearrangements constitute 40–70% of sporadic papillary carcinomas diagnosed in children and young adults (18). Regarding association with HT, RET/PTC rearrangement was more frequently observed in PTC associated with HT than in PTC without HT (31 vs. 13%, *P* = 0.02) (38). It was found that RET/PTC rearrangements were correlated with high TSH levels (*P* = 0.037) (41). Moreover, thyroid cell lines expressing RET/PTC may induce genes encoding molecules involved in the immune response (42), including *CXCL10*, which plays an important role in the first steps of HT lymphocytic infiltration (43, 44). Finally, it was reported that RET/PTC could be found both in areas of PTC and in areas with classic histological thyroid modifications typical of HT (45). Other genes have been theoretically implicated in the association between HT and thyroid cancer. *Human 8-oxoguanine glycosylase* is one of these genes. Mutations of this gene have commonly been found in both PTC (94%) and HT (73%), but not in other thyroid diseases (8%) (46). However, also in this case, children are different from adults because rearrangements appear more common in children, whereas mutations are more frequently detected in adults (46).

Independently of mutations in proto-oncogenes implicated in PTC, it seems likely that other genetic alterations may play a role in favoring the association between HT and thyroid cancer. This is suggested by the description of some clinical reports regarding children with thyroid cancer and HT associated with other autoimmune diseases, such as type 1 diabetes (47) and the autoimmune polyglandular syndrome type II (48).

## Conclusion

Despite its rarity and relatively promising prognosis, thyroid cancer remains a significant clinical problem in pediatrics. Its association with HT, despite being based on a significantly lower number of reliable studies than in adults, seems likely. However, although not all reasons for the coexistence of these diseases are clearly defined, children with HT should be considered at highest risk of cancer development. Strict correlations between high levels of serum TSH and anti-thyroid antibodies must be remembered. The same is true for the presence of nodules, especially if multiple nodules are present, Ultrasonography and TFNAC can favor an early identification of patients with malignant changes and should be promptly used in uncertain cases.

## Author contributions

LP conceptualized the work and wrote the first draft of the manuscript. MC, LL, and AL performed the literature analysis. NP gave a significant contribution of the event. SE supervised the work and gave a substantial scientific contribution. All the authors approved the final report.

### Conflict of interest statement

The authors declare that the research was conducted in the absence of any commercial or financial relationships that could be construed as a potential conflict of interest.
